# Unveiling tumor immune evasion mechanisms: abnormal expression of transporters on immune cells in the tumor microenvironment

**DOI:** 10.3389/fimmu.2023.1225948

**Published:** 2023-07-21

**Authors:** Lu Chen, Yuchen Wang, Qingqing Hu, Yuxi Liu, Xuchen Qi, Zhihua Tang, Haihong Hu, Nengming Lin, Su Zeng, Lushan Yu

**Affiliations:** ^1^ Institute of Drug Metabolism and Pharmaceutical Analysis, College of Pharmaceutical Sciences, Zhejiang University, Hangzhou, China; ^2^ Key Laboratory of Clinical Cancer Pharmacology and Toxicology Research of Zhejiang, Department of Clinical Pharmacy, Affiliated Hangzhou First People’s Hospital, Cancer Center, Zhejiang University School of Medicine, Hangzhou, China; ^3^ Center for Clinical Pharmacy, Cancer Center, Department of Pharmacy, Zhejiang Provincial People's Hospital (Affiliated People's Hospital), Hangzhou Medical College, Hangzhou, Zhejiang, China; ^4^ The Fourth Affiliated Hospital, School of Medicine, Zhejiang University, Jinhua, China; ^5^ Department of Neurosurgery, Sir Run Run Shaw Hospital, Zhejiang University School of Medicine, Hangzhou, Zhejiang, China; ^6^ Department of Pharmacy, Shaoxing People’s Hospital (Shaoxing Hospital, Zhejiang University School of Medicine), Shaoxing, China; ^7^ Westlake Laboratory of Life Sciences and Biomedicine of Zhejiang Province, Hangzhou, China; ^8^ Department of Pharmacy, Second Affiliated Hospital, School of Medicine, Zhejiang University, Hangzhou, China

**Keywords:** tumor immune evasion, transporters, metabolites, the tumor microenvironment, cancer therapy

## Abstract

The tumor microenvironment (TME) is a crucial driving factor for tumor progression and it can hinder the body’s immune response by altering the metabolic activity of immune cells. Both tumor and immune cells maintain their proliferative characteristics and physiological functions through transporter-mediated regulation of nutrient acquisition and metabolite efflux. Transporters also play an important role in modulating immune responses in the TME. In this review, we outline the metabolic characteristics of the TME and systematically elaborate on the effects of abundant metabolites on immune cell function and transporter expression. We also discuss the mechanism of tumor immune escape due to transporter dysfunction. Finally, we introduce some transporter-targeted antitumor therapeutic strategies, with the aim of providing new insights into the development of antitumor drugs and rational drug usage for clinical cancer therapy.

## 1 Introduction

The tumor microenvironment (TME) is a complex ecosystem that supports tumor cell survival and development. Tumor cells constitute the majority of the TME, along with peripheral blood vessels, stromal cells, extracellular matrix, and the secretion products (such as cellular metabolites and cytokines) of various cells (1). As a fundamental component of stromal cells, immune cells are indispensable for inhibiting the occurrence and development of tumors.

Tumor immune escape refers to the process whereby tumor cells escape the immune system’s “surveillance” instead of being eliminated, enabling them to proliferate and divide rapidly. The mechanisms of tumor immune escape can be divided into two main categories. One is that the TME suppresses antitumor immune responses by altering the function of immune cells, and the other is that tumor cells avoid being attacked by the immune system by decreasing the expression levels of self-antigens, creating defects in the antigen presentation machinery, or reducing their immunogenicity (2).

Transporters are membrane-binding proteins that mediate the passage of substrates through biological membranes. Transporters play integral roles in the cellular uptake of nutrients and the efflux of metabolic waste. Therefore, changes in the expression levels of transporters regulate the growth and function of cells. The TME has been found to affect the expression of transporters in immune cells, which can impair the ability of immune cells to kill tumor cells and control tumor progression.

This review provides an overview of the reciprocal regulatory mechanisms among metabolites in the TME, transporters in immune cells, and immune cell functions. In addition, we discuss potential cancer treatment regimens that target transporters to modulate immune responses, thus laying the groundwork for more efficient and safe antitumor therapies.

## 2 The tumor microenvironment and immune cells

### 2.1 Metabolic characteristics of the tumor microenvironment

The oxidative decomposition of glucose provides the primary energy source for cells. Normal cells acquire energy by thoroughly oxidizing glucose to CO_2_ and water under aerobic conditions, whereas tumor cells prefer to convert glucose into lactate via glycolysis, even under oxygen-sufficient conditions. This is referred to as the Warburg effect (3). Compared with oxidative phosphorylation (OXPHOS), aerobic glycolysis results in inefficient, but rapid, ATP production, accompanied by the production of intermediates and metabolites that act as major raw materials for cellular biosynthesis (4, 5). Collectively, aerobic glycolysis is conducive to the rapid growth and proliferation of tumor cells. Notably, the Warburg effect is not an exclusive feature of tumor cell metabolism, because cells in different tumor tissue regions preferentially utilize distinct metabolic pathways depending on the TME conditions and nutrient availability (6). Previous research has shown that oxygenated tumor cells produce large amounts of ATP through the oxidative phosphorylation of glucose, whereas hypoxic tumor cells mainly utilize glycolysis to supply energy and produce lactate for oxygenated tumor cells to fuel oxidative metabolism (7). This coupled metabolic pattern is of great significance for tumor cell growth and proliferation.

Glucose deficiency is a striking feature of the TME. The rapid proliferation of tumor cells enhances glucose uptake through the overexpression of the glucose transporters, GLUT1 and GLUT3, to relieve glucose dependence, resulting in glucose deficiency in the TME. It has been suggested that upregulated expression levels of glucose transporters in tumor cells is closely associated with poor cancer prognosis (8). Increased oxygen consumption resulting from tumor cell hyperproliferation creates a hypoxic microenvironment that contributes to tumor invasion and metastasis (9). In addition, both aerobic glycolysis and glutamine catabolism in tumor cells produce large amounts of lactate, rendering the TME acidic (10). Although most cancer-metabolism-related studies have considered lactate as a metabolic waste product in the Warburg effect, recent studies have suggested that lactate is a potential fuel (11).

Lipids are another abundant metabolite in the TME, and their accumulation is closely related to immune cell dysfunction and tumor cell survival (12). Amino acids are primarily involved in cellular component synthesis and energy metabolism (such as the TCA cycle), which, along with their metabolites, contribute to various effects on antitumor immune responses and tumor growth in the TME (13). Moreover, the high accumulation of nucleosides (especially ATP) in the TME contributes to tumor immune escape (14, 15).

In general, the characteristics of the TME, including hypoxia, acidification, lack of nutrients (such as glucose and amino acids), and massive accumulation of immunosuppressive metabolites (such as lactate, lipids, and nucleosides), effectively promote tumor progression and influence antitumor immune responses.

### 2.2 Functions of immune cells in the TME

Immune cells in the TME are divided into three categories based on their functions in tumor cells. The first group comprises immune cells with antitumor effects, mainly CD8^+^ T cells, CD4^+^ Th1 cells, natural killer (NK) cells, and dendritic cells (DCs). The second group consists of tumor-promoting immune cells, including CD4^+^CD25^+^ T cells (Tregs), myeloid-derived suppressor cells (MDSCs), and mast cells. Additionally, highly heterogeneous macrophages and neutrophils exert distinct immune functions via phenotypic changes in different local tissue microenvironments.

T cells are essential immune cells that can differentiate into CD8^+^ T cells with cytotoxic activity and CD4^+^ T cells with helper functions. CD8^+^ and CD4^+^ T cells have multiple cell subtypes that play multifaceted roles in tumorigenesis according to the different cytokines they produce (**Table 1**). The infiltration of CD8^+^ T cells and CD4^+^ Th1 cells at the tumor site is a hallmark of a favorable tumor prognosis (51), due to their production of the cytokines IFNγ and TNFα, which can effectively induce the cell cycle of tumor cells to arrest in the G1/G0 phase (20, 52). As the most powerful antigen-presenting cells, DCs play key roles in innate and adaptive immunity, as they can effectively activate resting T cells to transform them into cytotoxic T lymphocytes to promote an antitumor immune response (53). NK cells are also an integral part of the antitumor immune response. They exert cytotoxic effects on their target cells by forming immune synapses and directionally secreting lysed particles, such as perforin and granzyme (54).

**Table 1:** T cell subsets and functions.

T cell types	cell subsets	Surface antigens	Cytokines	Antitumor reactivity	References
CD4^+^ T cell	Treg	CD25、CTLA-4	IL-10, IL-35, TGFβ	Negative	(16–19)
	Th1	CXCR3、 CCR5	IFNγ, TNF	Positive	(20, 21)
	Th2	CCR4、CCR8、CRTH2	IL-3, IL-4, IL-5, IL-13	IL-3, IL-5, IL-4: Positive IL13: Negative	(22–28)
	Th9	CXCR3、CCR3 CCR6	IL-9, IL10	Positive/Negative	(28)
	Th17	CCR4、CCR6	IL-17A, IL-17F, IL-22	IL-17A, IL-17F: Positive/Negative IL-22: Negative	(27, 29–36)
	Th22	CCR4、CCR6、CXCR10	IL-22	Negative	(27, 36)
	Tfh	CD40L、PD-1、CXCR5	IL-4, IL-21	IL-4: Negative IL-21: Positive	(37–41)
CD8^+^ T cell	Tc1	IL18R	IFN-γ, TNF-α	Positive	(42–44)
	Tc2	CRTH2	IL-4, IL-5, IL-13	Positive	(44–46)
	Tc9	CCR6、PD-1	IL-9	Positive	(47, 48)
	Tc17	CD86、CD101	IL-17A, IL-17F, IL-21, IL-22	Negative	(43, 49, 50)
	Tc22	ICOS、4-1BB	IL-2, TNF-α, IL-22	Positive	(50)

Tregs inhibit autoimmune reactions and maintain immune homeostasis *in vivo* (55). Tregs expressing the transcription factor Foxp3 are closely associated with a poor prognosis in multiple cancers (56). Similarly, Th2 and Th17 cells contribute to tumor occurrence and development (51). MDSCs are myeloid-derived cells, including polymorphonuclear MDSCs (PMN-MDSCs) and mononuclear MDSCs (M-MDSCs) (57). Tumor-infiltrating MDSCs exert tumor-promoting effects by inducing the generation of immunosuppressive Tregs and M2-type macrophages and inhibiting T cell activation (58). Mast cells, also known as basophils, are derived from myeloid cells and they secrete several bioactive molecules with tumor-promoting functions, such as angiogenic factors and matrix metalloproteinases (59). In addition, IL-33-activated mast cells promote tumor outgrowth by secreting macrophage chemokines to facilitate tumor-associated macrophages (TAMs) recruitment to tumor sites (60).

Macrophages and neutrophils perform distinct functions through environment-dependent phenotypic transformations. It is generally believed that macrophages mainly fall into two categories: type 1 (M1) and type 2 (M2) macrophages. M1 macrophages with proinflammatory and tumor-suppressive effects primarily rely on aerobic glycolysis for their energy supply, whereas M2 macrophages utilize fatty acid oxidation (FAO) to fuel mitochondrial OXPHOS, which is beneficial for repairing tissues, maintaining metabolic homeostasis, and enhancing tumor progression (61). Tumor cells recruit TAMs with an M2-like phenotype to the tumor microenvironment to disrupt immune surveillance (62). Similar to TAMs, tumor-associated neutrophils (TANs) can be divided into an N1 phenotype with antitumor activity and an N2 phenotype with protumor activity. The immunosuppressive cytokine TGF-β, which is overexpressed by tumor cells, can induce TAN polarization toward the N2 phenotype (63).

### 2.3 Effects of metabolites on immune cell function in the TME

#### 2.3.1 Lipids

Lipids in the TME are pivotal for the suppression of antitumor immune responses. Lipids reduce CD8^+^ T cell cytotoxicity by inducing lipid peroxidation and ferroptosis (64, 65). Cholesterol accumulation in tumor-infiltrating CD8^+^ T cells is also closely related to the increased expression levels of immune checkpoint factors and cell exhaustion (66). Excessive intracellular lipids in DCs damage their antigen-presenting function and further inhibit T cell priming in the TME (67, 68). In NK cells, high lipid levels lead to a decrease in IFN-γ levels and the induction of metabolic reprogramming, thus significantly blunting their cytotoxic effects on tumor cells (69). The TME induces a phenotypic switch from M1 to M2 macrophages with lipid dependency. Lipid accumulation in TAMs is required for cell differentiation and tumor-promoting function (70). In addition, lipid-overloaded MDSCs have a stronger immunosuppressive effect (71).

#### 2.3.2 Glucose

Glucose is the major nutrient that fuels cellular metabolic activity. Competition between tumor cells and T cells for glucose leads to the exhaustion of tumor-infiltrating T lymphocytes in the TME (72). At the same time, T cells derived from the TME have metabolic defects that manifest as decreased expression levels of glucose transporters and metabolic enzymes, which are detrimental to antitumor immune responses (73, 74). Because the increased metabolic demands for glucose and glutamine in activated T cells cannot be satisfied in the nutrient-deficient TME, protein glycosylation, cell differentiation, and growth of T cells are maintained (75, 76). Activated NK cells regulate metabolic reprogramming toward glycolysis via mTORC1, to adapt to the glucose-deficient TME (77). Similarly, Tregs inhibit glycolysis by highly expressing Foxp3 to achieve better metabolic adaptation to the low-glucose TME (78). Tumor-derived exosomes (TDEs) endow TAMs with immunosuppressive phenotypes by enhancing glucose uptake and metabolism (79).

#### 2.3.3 Lactate

Excess lactate excretion improves tumor progression by enhancing acidification and regulating multiple signaling pathways in different cells (9). Lactate accumulation in the TME limits its efflux in a concentration-dependent manner and promotes the expression of lactate-uptake transporters, resulting in elevated levels of intracellular lactate, which interfere with cell metabolism and immune function (80, 81). Similarly, lactate is essential for the maintenance of Treg activity. Tumor-infiltrating Tregs exhibit upregulated expression levels of lactate-uptake- and metabolism-related genes to acquire adaptability and exert their immunosuppressive function (82). Additionally, in an environment with high lactate concentrations, macrophages are prone to M2 polarization, whereas the maturation of DCs is attenuated. These findings are consistent with immunosuppression generated by the TME (83).

#### 2.3.4 Amino acids

Amino acids are closely associated with tumor development, and amino acid deficiency in the TME typically triggers immune cell dysfunction. Notably, the accumulation of certain amino acids, such as kynurenic acid (Kyn), are closely correlated with tumor development and progression. Kyn-mediated AHR signal conduction is a key mechanism that regulates the interaction between Tregs, TAMs, and CD8+ T cells to endow the TME with immunosuppressive properties (84–88). Branched-chain amino acids (BCCAs) are essential contributors to tumor growth. Upregulation of the BCCA transaminase 1, which is involved in BCCA catabolism, has been observed in various tumors (89). The accumulation of the BCCA metabolites, branch chain keto acids (BCKAs), excreted by glioblastoma cells via monocarboxylate transporter 1 (MCT1), suppresses antitumor immune responses by attenuating the phagocytic activity of macrophages (90).

#### 2.3.5 Nucleosides

Hypoxic conditions induce ATP accumulation in the TME. Extracellular ATP can be rapidly degraded into adenosine, an immunosuppressive effector that participates in key signaling pathways that regulate tumor immune responses (15). In lymphocytes, signal transduction between adenosine and the A2A adenosine receptor inhibits cytotoxic T cell activation, while favoring Treg proliferation and immunosuppressive activity (91, 92). Regarding myeloid cells, adenosine enhances the IL-10-induced activation of M2 macrophages and PMN-MDSC expansion (93, 94). The ectonucleoside triphosphate diphosphohydrolase, ENTPD2, which is highly expressed in many types of tumors, hydrolyzes extracellular ATP into 5’-AMP to maintain the immunosuppressive function of M-MDSCs (95).

## 3 Effects of metabolic transporters on immune cells in the tumor microenvironment

### 3.1 Lipid transporters

**Figure 1:**
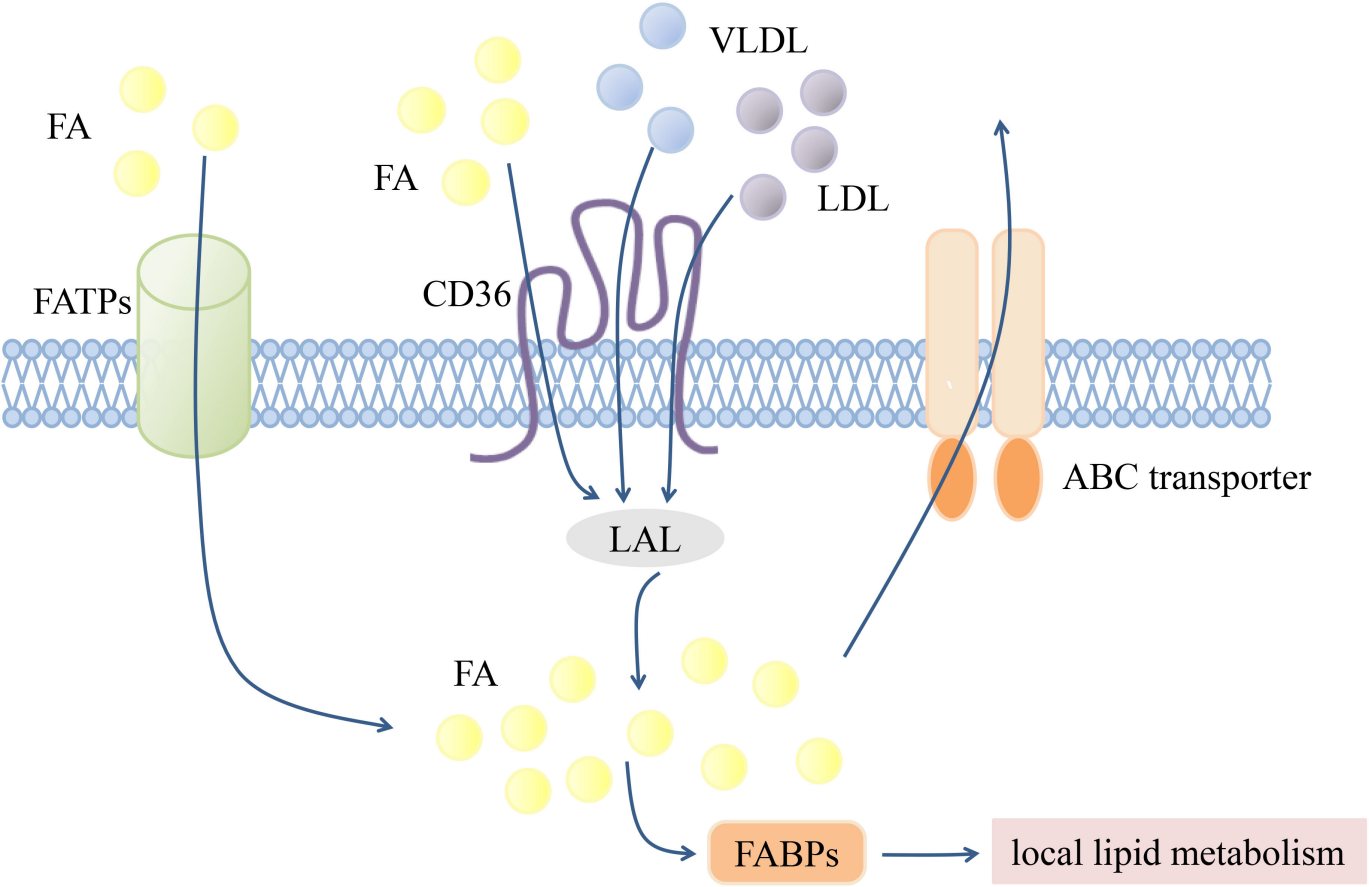
Transmembrane transportation of lipids. Transmembrane transport of lipids is mediated by various transport proteins. FATPs and CD36 are mainly responsible for mediating exogenous FA uptake. LDL and VLDL can also achieve intracellular transport through CD36, and then hydrolyzed by LAL into FA. Intracellular FA can be transported to different organelles or compartments for subsequent local lipid metabolism by FABPs, or excreted by ABC transporters. Fatty acid (FA), The fatty acid transporter family (FATPs), The fatty acid binding proteins (FABPs), Lysosomal acidic lipase (LAL), Low-density lipoprotein (LDL), Very low-density lipoprotein (VLDL).

According to the “Comprehensive Classification System for Lipids” published by the International Lipid Classification and Nomenclature Committee in 2005, lipids can be divided into the following eight categories: fatty acyls, glycerolipids, glycerophospholipids, sphingolipids, sterol lipids, prenol lipids, saccharolipids, and polyketides (96). Since lipids are cellular nutrients and signaling molecules, functional changes in lipid transporters are key modulators of the immune response. Several transmembrane and intracellular transporters mediate lipid transport (**Figure 1**). The CD36 transporter and fatty acid transport proteins (FATPs) are major lipid uptake transporters. Lipids are ingested intracellularly by the CD36 transporter in the form of low-density lipoprotein (LDL), very-low-density lipoprotein (VLDL), and fatty acids (61, 70). The FATP family, which is composed of six members (FATP1-6), mediates exogenous fatty acid uptake with different tissue expression patterns (97). Fatty acid binding proteins (FABPs) are a family of small proteins that act as intracellular fatty acid transporters (98), while ABCA1 and ABCG1 can synergistically mediate intracellular cholesterol efflux (99).

#### 3.1.1 CD36

The scavenger receptor, CD36, which has a lipid transport function, is widely expressed in various types of immune cells. The TME promotes tumor cell proliferation by interfering with CD36-mediated lipid metabolism in immune cells. Increased CD36 expression levels in CD8^+^ T cells allows the accumulation of lipid peroxides in cells and leads to the decreased secretion of cytotoxic factors, causing the dysfunction of tumor-infiltrating CD8^+^ T cells (64, 65). CD36 also functions as a regulator of tumor-infiltrating Tregs. The CD36-PPARβ signaling pathway mediates mitochondrial fitness and NAD production to support Tregs, which are more adaptable to low glucose and high lactate levels (78, 100). M2 macrophage activation is dependent on FAO. M2 macrophages increase LDL and VLDL uptake by upregulating CD36 expression levels, followed by lysosomal acidic lipase hydrolysis to provide fatty acids as substrates for FAO (61). CD36 directly mediates fatty acid transport. Increased CD36 expression levels in TAMs is conducive to an immunosuppressive function and cancer progression by enhancing fatty acid absorption and FAO (70). As a crucial regulator of TAM polarization, S100A4 upregulates CD36 expression levels by acting on PPAR-γ to induce FAO and then drives the M2-like polarization of TAMs (101). Lipid accumulation associated with the upregulation of CD36 and CD204 in DCs reduces antigen processing by DCs, limiting T cell priming (67, 68). MDSCs increase exogenous lipid uptake by upregulating the expression of multiple lipid transporters involved in metabolic reprogramming to support their immunosuppressive functions (71, 102). CD36 upregulation is closely related to Pim1-PPARγ signal transduction by PIM1 kinase, thus enhancing lipid metabolism and the immunosuppressive function of MDSCs (103). Similarly, lipid accumulation in NK cells caused by high CD36 expression levels, also attenuates the toxic effects on cancer cells (104).

#### 3.1.2 The FATP/FABP family

FATPs encoded by the *SLC27A* family mediate the uptake of exogenous fatty acids and exhibit acetyl-CoA synthetase activity (105). Veglia et al. reported that the high expression levels of FATP2 (*SLC27A2*) in tumor-infiltrating PMN-MDSCs results in enhanced arachidonic acid uptake and prostaglandin E2 synthesis, thus endowing PMN-MDSCs with immunosuppressive activity (106). Subsequent studies by Adeshakin et al. showed that lipid accumulation in MDSCs induces immunosuppressive activity by enhancing mitochondrial function and activating reactive oxygen species (105).

FABPs encoded by *FABP1-9* genes are other key proteins involved in intracellular lipid transportation, with a high affinity for long-chain fatty acids (98). Previous studies have found that M2 macrophages preferentially upregulate FABP4 expression and play a tumor-promoting role through FABP4-dependent IL-6/STAT3 signaling (107). In contrast, FABP5 in M1 macrophages enhances the recruitment of tumor-killer immune cells, such as effector T cells and NK cells, to the tumor site by inducing IFN-β secretion, thus promoting antitumor immune responses (108). FABP5 has been implicated as a regulator of macrophage phenotypes, contributing to M2 polarization, and FABP5 deficiency is hostile to the anti-inflammatory response of murine macrophages (109). High FABP5 expression levels in tumor-infiltrating Tregs enhance mitochondrial disturbances, type I IFN signaling, and immunosuppressive activity (110).

#### 3.1.3 ATP-binding cassette transporters

ATP-binding cassette (ABC) transporters are members of the ABC protein superfamily. They couple ATP hydrolysis with substrate transport. As an integral component of the cell membrane structure, cholesterol is a key regulatory effector of the immune response. For example, T cell activation mediated by T cell receptor (TCR) signaling relies on intracellular cholesterol (111, 112). The ABC transporters ABCA1 and ABCG1 are the predominant cholesterol efflux transporters in immune cells. ABCG1 is a negative regulator of lymphocyte proliferation, and the loss of ABCG1 leads to enhanced TCR signaling in CD4^+^ T cells and promotes cell proliferation by promoting the accumulation of cholesterol (113). However, the effect of cholesterol on CD8+-T-cell-mediated antitumor immune responses remains controversial. The reduction in cholesterol uptake by CD8^+^ T cells induced by the TME inhibits TCR signaling and antitumor activity (114). Previous studies have also demonstrated that intracellular cholesterol negatively regulates the antitumor function of CD8^+^ T cells (66, 115). Accordingly, further studies are needed to determine the regulatory effects of cholesterol on T cell function.

ABCA1 and ABCG1 mediate cholesterol efflux from macrophages (99). Cholesterol metabolites and oxysterols inhibit T cell proliferation and immune effector capacity by activating LXR to induce ABCA1 and ABCG1 upregulation in macrophages (116). In ABCG1-deficient macrophages, cholesterol accumulation promotes proinflammatory gene expression mediated by NF-κB, thereby inducing macrophage polarization to the M1 phenotype (117). In ABCA1/G1-deficient DCs, accumulated cholesterol activates the DC inflammasome and promotes the secretion of inflammatory cytokines, leading to autoimmune diseases (118). ABCG1 deficiency is associated with the proliferation and maturation of invariant NK T (iNKT) cells (119). Additionally, ABCA7, another ABC family transporter in iNKT cells, regulates cellular functions by mediating the efflux of phospholipid complexes (120).

### 3.2 Glucose transporters

As a vital nutrient that supports energy production and biomass synthesis in cells, glucose is mainly transported into cells through GLUTs, a family of glucose transporters encoded by the *SLC2A* gene family (121). Activated CD4^+^ T cells support metabolic reprogramming toward aerobic glycolysis by upregulating GLUT1 expression levels to rapidly proliferate and differentiate into immune-promoting effector T (Teff) cells and immune-inhibiting Treg cells (122). Decreased GLUT1 expression levels in T cells from cancer patients may be closely related to the expression levels of the inhibitory receptors, PD-1 and TIM3 (73, 74, 123). A study of lung squamous cell carcinoma identified GLUT3 as the main glucose transporter in immune cells of the TME (124). Increased glucose uptake by GLUT3 in T cells inhibits GLUT3-mediated glucose uptake by cancer cells, indicating that immune cells with high GLUT3 expression levels promote effective antitumor immunity (124). Teff cells selectively depend on the glucose transporter GLUT1 for proliferation and inflammatory responses (122, 125). In addition, Tregs with increased GLUT1 expression levels and glycolytic reactions in the TME are functionally unaffected by GLUT1 deficiency, possibly because the energy supplementation pathway involves lipid metabolism (122, 126). Compared to normal neutrophils, neutrophils with pro-tumor functions exhibit higher GLUT1 expression levels and glucose metabolism (127). Similarly, TDE-induced macrophages achieve metabolic reprogramming by increasing the expression levels of GLUT1, HIF1-α, and lactose dehydrogenase A, which polarizes TAMs toward protumor phenotypes (79).

### 3.3 Lactate transporters

Monocarboxylate transporters (MCTs) belong to the SLC solute carrier family and comprise 14 members. MCT1–4 are proton-coupled monocarboxylate transporters capable of mediating the bidirectional transport of lactate, driven by a transmembrane concentration gradient (128). MCT1 and MCT4 are the most extensively studied monocarboxylate transporters involved in human cancers. MCT1 plays a major role in lactate uptake by glycolytic cells, whereas MCT4, which has a low affinity for lactate, is not easily saturated by high intracellular lactate levels and promotes lactate efflux in glycolysis-dependent cells, such as tumor cells (128, 129). High expression levels of MCT1 and MCT4 have been observed in many types of tumor cells (128). The Na-coupled transporters SMCT1 (SLC5A8) and SMCT2 (SLC5A12) also participate in lactate transport (129, 130).

Excess lactate accumulation in the TME inhibits the antitumor effects of immune cells. For activated T cells, increased lactate in the TME leads to an impaired lactate efflux function of MCT1, thus affecting glycolysis and the immune function of cytotoxic T cells (80). For NK cells, the downregulation of MCT4 expression levels in tumor cells can effectively restore their tumor-killing function by reversing the immunosuppressive TME (131). Conditions of high lactate concentration affect the antigenic phenotype and functional activity of monocyte-derived DCs, and induce MCT1 expression in plasma cell-like dendritic cells (pDCs), thereby facilitating tumor cells to evade immune surveillance (132, 133). Moreover, lactate inhibits the TLR stimulation response and IFN-α secretion through MCT1-mediated intracellular lactate transport and promotes pDCs to induce Tregs (133). Tregs with high MCT1 expression levels upregulate PD-1 expression and maintain their immunosuppressive activity in the glucose-deficient TME (82, 134). Additionally, lactate facilitates tumor immune escape by upregulating MCT1 expression levels in macrophages, thereby enabling M2 macrophage polarization (135, 136). Increased MCT4 expression levels in macrophages enhances lactate efflux, which is necessary for macrophage activation, glycolysis, and inflammatory responses (137).

To date, there has been no detailed investigation of the changes in SMCT expression levels in immune cells. It has been proposed that lactate accumulation upregulates SMCT2 expression levels in CD4^+^ T cells to enhance lactate uptake and the production of the proinflammatory cytokine IL-17, resulting in decreased glycolysis, cell activity, and migration (81, 138). DCs and macrophages also express *SLC5A8* and *SLC5A12*, but they do not appear to exert a lactate transport function (130, 139).

### 3.4 Amino acid transporters

**Figure 2:**
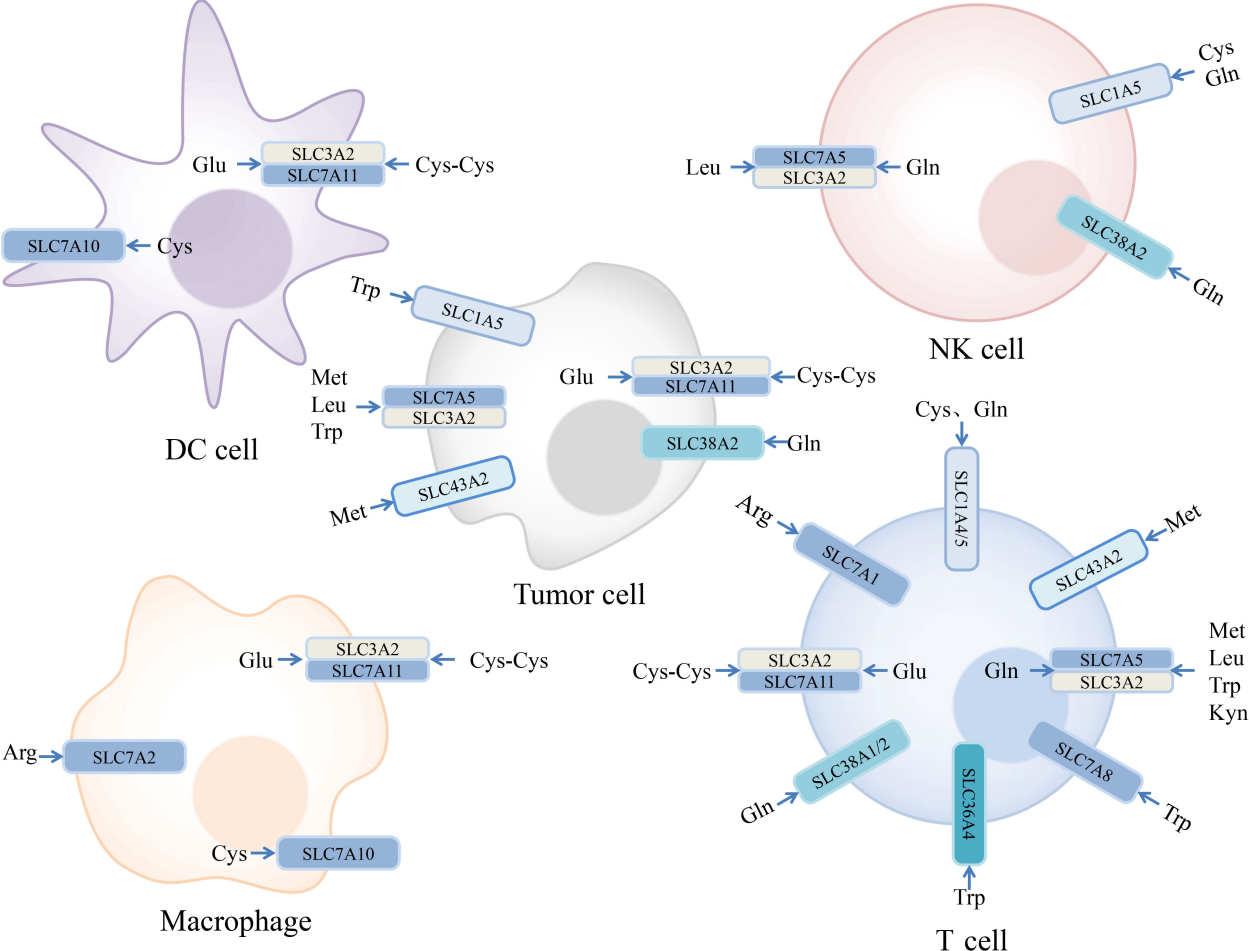
Expression of amino acid transporters in tumor and immune cells and their substrates. Some amino acids and their transporters are engaged in the regulation of the immune responses in the tumor microenvironment. Examples we mentioned are depicted in the figure. Arginine (Arg), Cysteine (Cys), Cystine (Cys-Cys), Glutamate (Glu), Glutamine (Gln), Kynurenine (Kyn), Leucine (Leu), Methionine (Met), Tryptophan (Trp).

Amino acids are involved in a series of complex physiological processes, such as protein synthesis, metabolism, and signal transduction. There are three major classes of amino acid transporters in the SLC superfamily. Based on their properties, amino acid transporters can be divided into neutral, basic, and acidic types. Depending on whether the transport process requires coupling with Na^+^, they can be further divided into Na^+^- and non-Na^+^-dependent transporters (13). When comprehensively considering substrate specificity and transport mechanisms, they can be further divided into multiple systems, such as A, N, ASC, B, L, T, x_c_^−^, and y^+^ (140). The relationship between amino acids and their transporters is not a one-to-one relationship (**Figure 2**), making research targeting amino acid transporters more challenging (13).

#### 3.4.1 Cysteine and cystine transporters

Typically, cystine is rapidly reduced to cysteine, which is necessary for the formation of the antioxidant glutathione (GSH) in cells (141). System x_c_^−^, which is composed of xCT (*SLC7A11*) and CD98 (*SLC3A2*) subunits, is a major plasma membrane antiporter responsible for the cellular uptake of cystine in exchange for intracellular glutamate (142). NaïveT cells cannot utilize cystine and methionine to synthesize cysteine because of a lack of cystathionase and complete x_c_^−^ transporters. In this setting, T cell activation requires the neutral amino acid transporters ASCT1 (*SLC1A4*) and ASCT2 (*SLC1A5*) for the cellular uptake of cysteine excreted by macrophages and DCs through ASC transporters (*SLC7A10*) (143). Activated T cells upregulate the expression levels of members of the x_c_^−^ system and neutral amino acid transporters to ingest cysteine for proliferation, thus deviating from a dependency on extracellular cysteine (144, 145). Activated monocytes and macrophages also ingest cystine mainly through the x_c_^−^ system and upregulate x_c_^−^ component expression levels in response to inflammatory stimulation to exercise immune functions (146, 147). The highly expressed x_c_^−^ system in mature DCs plays a significant role in mediating the differentiation of monocytes into DCs (148). MDSCs deplete cysteine in the TME and block T cell activation by rendering DCs and macrophages unable to support T cells (143). CD8^+^ T cells induce lipid peroxidation and ferroptosis through the downregulation of *SLC3A2* and *SLC7A11* levels in tumor cells by secreting IFN-γ, thereby mediating effective antitumor responses (149). Elevated expression levels of *SLC7A11* in tumor cells contribute to tumor invasion and metastasis, which are also correlated with poor cancer prognosis (150). Although *SLC7A11* deletion in tumor cells results in impaired tumor growth, neither systemic nor T-cell-specific knockout of *SLC7A11* affects T cell proliferation or the antitumor immune response, suggesting that further investigation of the interplay between *SLC7A11* and T cell function should be conducted (13, 151).

#### 3.4.2 Glutamine transporters

Glutamine is a nonessential amino acid that regulates vital cellular activities, such as energy metabolism and biosynthesis. As the deaminated form of glutamine, glutamate is further converted into ketoglutarate, which enters the TCA cycle, where it serves as a substrate for fatty acid, amino acid, and nucleotide synthesis (152, 153). The amino acid transporter system L, composed of LAT1 (*SLC7A5*) and CD98 (*SLC3A2*), exchanges glutamine for essential amino acids to regulate cell proliferation and function, indicating that intracellular glutamine is vital for amino acid transport (154–157). Glutamine is indispensable for T cell activation; therefore, most studies on glutamine transporters in immune cells have focused on T cells. TCR and CD28 activate T cells by stimulating the downstream MAPK family member, ERK, to induce the expression of the glutamine transporters SNAT1 (*SLC38A1*) and SNAT2 (*SLC38A2*). SNAT1 displays higher expression levels because of its faster induction (153). Similarly, ASCT2-mediated glutamine uptake plays a constructive role in T cell activation mediated by the mTORC1 signaling pathway. Therefore, ASCT2 deficiency suppresses the inflammatory response by inhibiting CD4^+^ T cell differentiation into Th1 and Th17 cells (155). LAT1 deletion also inhibits CD4^+^ T cell expansion and decreases the secretion of inflammatory factors (158). Amino acid transport mediated by ASCT2 and transporter system L is crucial for maintaining cellular metabolism and function in NK cells by regulating cMyc expression (154, 159).

#### 3.4.3 Methionine transporters

Methionine is a sulfur-containing proteinogenic amino acid required for the synthesis of spermine and the major reducing agent GSH (160). In addition, methionine serves as the only source of the intracellular universal methyl donor S-adenosylmethionine (SAM), which plays an integral role in regulating gene expression and basic metabolic pathways in cells (161). Activated T cells increase methionine uptake by upregulating SLC7A5 expression levels and producing methyl donors for DNA and RNA methylation (162). In a hypoxic microenvironment, upregulated SLC7A5 expression contributes to the accumulation of methionine in tumor cells, which leads to methionine depletion in the TME (163). Therefore, decreased methylation in T cells may be attributed to an insufficient level of methionine, which affects cell proliferation and function (164). Since another methionine transporter encoded by *SLC43A2* is also highly expressed in tumor cells, tumor-infiltrating effector T cells with low SLC43A2 expression levels do not compete with tumor cells for methionine. Consequently, low methionine and SAM levels and weakened H3K79me2 signaling in the promoter region downregulate STAT5 expression, which in turn affects T cell survival and function (165). Tumor cells escape immune surveillance by inducing T cell exhaustion through the cellular methionine metabolites SAM and 5-methylthioadenosine (166).

#### 3.4.4 Leucine transporters

Transport of the essential branched-chain amino acid, leucine, and other large neutral amino acids is predominantly mediated by the amino acid transporter system L (167, 168). The mTORC1 signaling pathway is crucial for T cell activation and differentiation. Previous studies have shown that T cells upregulate *SLC7A5* expression under sustained immune activation via TCR or IL-2 to improve leucine uptake and activate mTOR signaling (168). Notably, leucine uptake mediated by system L must be coupled with glutamine efflux, which is dependent on ASCT2-mediated glutamine uptake (169). This conclusion was confirmed in a follow-up study in which Nakaya et al. effectively activated the mTORC1 signaling pathway in *SLC1A5-*deficient T cells by exogenous supplementation with leucine (155). The inhibition of *SLC7A5* expression affects the NK cell effector function, resulting in lower C-MYC protein expression levels and mTORC1 signaling in NK cells (167). Overexpression of LAT1 is also closely related to the proliferation of cancer cells, such as lymphoma, esophageal cancer, lung cancer, and prostate cancer cells (156), owing to the critical role of *SLC7A5* in activating mTORC1 signaling and metastasis in tumors (156, 170).

#### 3.4.5 Tryptophan and kynurenine transporters

Tryptophan metabolism through indoleamine 2,3-dioxygenase (IDO) and tryptophan 2,3-dioxygenase (TDO), which are involved in the kynurenine pathway, is postulated to be a leading cause of tumor immune escape (171). Tryptophan is transported via two pathways. One is the amino acid transporter system L, which is ubiquitously expressed in mammalian cells, and is the only conventional transport system expressed by resting human T cells. The other is a novel transport system upregulated by IDO in tumor cells and human monocyte-derived macrophages, with a high affinity for tryptophan (172, 173). Tumor cells upregulate the expression of multiple transporters that rely on the transcription factor ATF4 to enhance tryptophan and glutamine uptake under IDO-induced tryptophan deficiency (171). Moreover, overexpression of the tryptophan-transport- and metabolism-related genes *SLC1A5, SLC7A5, SLC7A8*, and *TDO2* in tumor cells also provides potential strategies for cancer therapy (174, 175). *SLC1A5* expression is also upregulated in a highly tumorigenic subpopulation of tumor cells known as tumor-repopulating cells (175). Correspondingly, enhanced tryptophan uptake and metabolism in tumor cells leads to kynurenine accumulation in the TME, thereby dampening antitumor immune responses. Extensive research has shown that kynurenine transport in activated T cells is mediated by transporters encoded by *SLC7A5*, *SLC7A8*, and *SLC36A4* genes (175, 176). As LAT1 simultaneously mediates the transport of multiple amino acid substrates, high levels of kynurenine in the TME exhibit competitive inhibition with other substrates, thus providing a new explanation for the immunosuppressive mechanism mediated by kynurenine accumulation in the TME (176).

#### 3.4.6 Arginine transporters

The conditionally essential amino acid arginine is the main substrate for the biosynthesis of proteins, creatine, nitric oxide, and polyamines, and is involved in activating the metabolic checkpoint, mTOR, for cell proliferation (177, 178). Arginine uptake is mainly mediated by transporter system y (CAT1-3), encoded by the *SLC7A1-3* gene (179). Arginine metabolism is essential for sustaining tumor metabolism and survival (180, 181). CAT1 is the only transporter responsible for L-arginine uptake in chronic lymphocytic leukemia (CLL) cells. Previous studies have shown that downregulating CAT1 expression in mouse CLL cells has a significant tumor-inhibition effect (182). Therefore, CAT1 may serve as a novel therapeutic target for CLL treatment. Similarly, arginase produced by tumor cells and MDSCs inhibits T cell proliferation and leads to tumor immune escape (183–185). In addition to arginine, creatine and polyamines also have immunomodulatory properties. The upregulation of CAT1 in activated T cells results in increased L-arginine uptake, which contributes to T cell proliferation and tumor-suppressive functions (186, 187). Polyamine metabolism affects transcriptome and epigenome remodeling from Th17 cells to Tregs (188). The expression of CAT2 is induced in both M1 and M2 macrophages (189). Additionally, macrophages ingest creatine, a metabolite of arginine, in a process mediated by the transporter CRT (*SLC6A8*), to polarize them toward the M2 phenotype (190).

### 3.5 Nucleoside transporters

Equilibrative nucleoside transporters (ENT1–4) encoded by the *SLC29* family and condensed nucleoside transporters (CNT1-3) encoded by the *SLC28* family are the major nucleotide transporters in the human body. ENTs have been implicated in the bidirectional transport of various purines and pyrimidines to maintain intracellular and extracellular nucleoside homeostasis. CNTs mediate the unidirectional uptake of nucleosides in an Na^+^- and H^+^-coupled manner (191, 192). Nucleoside transporters play a central role in transporting nucleosides, nucleobases, and nucleoside analogs, with effects on cell metabolism and signal transduction (192–194). Several studies have suggested that decreased ENT1 and CNT1 expression levels in tumor cells may be associated with drug resistance (192, 193, 195, 196). In addition, the RFC1 transporter encoded by *SLC19A1* mediates the uptake of the immune transmitter cGAMP into monocyte-derived U937 cells to elicit antitumor immune responses via immune cell recruitment. However, whether other immune cells utilize this mechanism to achieve cGAMP uptake requires further investigation (197).

Hypoxia is a common feature of the TME, with inhibitory effects on cellular ENT1 expression (198–200). For example, nucleotide metabolic enzymes CD73 and CD39, expressed by immune cells, are upregulated under the regulation of HIF1-α. This leads to adenosine accumulation in the TME, which negatively affects the immune activity of Teff cells (198, 199, 201). In mice with inflammatory diseases, the ENT1-targeted drug decitabine selectively depletes Teff cells with high ENT1 expression levels and promotes the proliferation of immunosuppressive Treg cells, which may be one of the mechanisms responsible for immune tolerance (202). Macrophages simultaneously express ENTs and CNTs, and selectively express specific nucleoside transporters according to the requirements related to their growth, development, and metabolism, with regulators including lipopolysaccharide, TNF-α, IFN-γ, and M-CSF (203, 204).

## 4 Tumor therapeutic regimens targeting transporters

**Figure 3:**
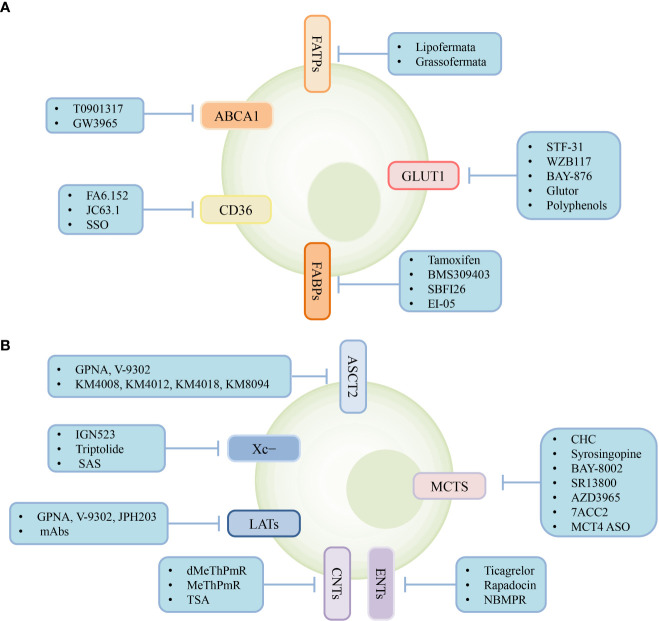
Transporter-targeted antitumor drugs. Current approaches including chemical drugs and antibodies for transporter-targeted antitumor drugs. **(A)** Lipid and glucose transporters-targeted drugs. **(B)** Lactate, amio acid and nucleoside transporters-targeted drugs.

Metabolic reprogramming of tumor and immune cells occurs owing to the rapid proliferation of tumor cells, ultimately creating an immunosuppressive TME with complex interactions between metabolite abundance and transporter expression. Therefore, targeting transporters expressed in the cells of the TME to regulate cellular metabolism may provide powerful support for cancer treatment. Various types of transporter-targeted antitumor drugs have been developed, including chemical drugs and antibodies (**Figure 3**). In addition, the combination of transporter-targeted drugs with existing chemotherapeutic drugs or immune checkpoint inhibitors may provide new strategies for clinical cancer treatment.

### 4.1 Targeting lipid transporters

The irreversible CD36 inhibitor sulfo-n-succiniminooleate (SSO) inhibits the uptake of oxidized low-density lipoprotein by macrophages (205). Experimental studies in mice have shown that the CD36-neutralizing antibodies FA6.152 and JC63.1 hold promise for inhibiting cancer cell metastasis in various cancer types (206).

Lipofermata and grassofermata specifically inhibit FATP2-mediated fatty acid transport and cytotoxic reactions caused by excessive fatty acid accumulation (106, 207). Moreover, FATP2 inhibitors, in combination with checkpoint inhibitors, alleviate the suppressive activity of MDSCs against T cells and significantly enhance antitumor immune responses (105, 106). Lipofermata may also be exploited to inhibit FATP1 because of the similarity between FATP1 and FATP2, which contributes to the effective inhibition of FATP1-mediated fatty acid uptake, thereby reducing melanoma cell proliferation (208). However, lipofermata shows no obvious therapeutic effects in tumor-bearing mice owing to its short half-life (208). Pharmacological inhibitors of FABP4, such as tamoxifen and BMS309403, effectively inhibit tumor progression (209–211). The chemical inhibitor SBFI26 restricts tumor proliferation by targeting FABP5-PPARγ-VEGF signaling (212). High FABP5 expression levels confer enhanced antitumor activity to macrophages. The small-molecule compound EI-05, identified by Rao et al., significantly increase FABP5 expression levels in macrophages and inhibits breast tumor growth in mice (213).

The ABCA1 transporter is also a potential target for tumor treatment. ABCA1 suppresses tumor growth by mediating cholesterol efflux in tumor cells to promote cell death (214, 215). Missense mutations in ABCA1 contribute to tumor progression in patients with chronic myelomonocytic leukemia (216). The tumor suppressor P53 induces ABCA1 expression, and loss of P53 or ABCA1 promotes liver tumor cell proliferation in mice (217). As the target gene of the nuclear receptor LXR, ABCA1 expression can be increased by LXR activation. Therefore, the LXR agonists T0901317 and GW3965 play crucial roles in the anti-proliferative effects on tumor cells (218). LXR agonists combined with first-line therapeutic drugs for melanoma, such as dacarbazine, vemurafenib, and anti-CTLA-4 antibodies, improve the clinical efficacy of monotherapies (219). Similarly, the combination of GW3965 and gemcitabine exhibits a more significant antitumor effect than two single-agent treatments on pancreatic cancer cell lines (220).

### 4.2 Targeting glucose transporters

Treatments targeting glucose transporters mainly include drug therapies targeting GLUT1 and those providing RNA interference. Compound STF-31, identified by Chan et al., exhibits synthetic lethality in cancer cells with high GLUT1 expression levels and glycolysis dependence (221). WZB117, a small-molecule inhibitor, exerts anticancer effects by reducing GLUT1 expression levels, intracellular ATP levels, and glycolytic enzyme levels in tumor cells (222). BAY-876 is a highly selective inhibitor of GLUT1 with favorable pharmacokinetic properties both *in vitro* and *in vivo* and it exhibits potent antitumor effects against a variety of solid tumors (223–226). Polyphenols, such as resveratrol, hesperetin, catechin, and quercetin, also possess antitumor functions by reducing GLUT1 mRNA and protein expression levels in tumor cells (227). The growth of tumor cells treated with an anti-*SLC2A1* short hairpin RNA (shRNA) is markedly suppressed (228). Furthermore, an shRNA targeting *GLUT1* enhances cisplatin sensitivity in head and neck cancer cells (229). Collectively, RNA combined with chemotherapeutic drugs is expected to result in improved therapeutic effects against cancer. Notably, in addition to GLUT1, tumor cells upregulate GLUT3 expression levels under conditions of glucose deprivation. Therefore, drugs specifically targeting GLUT1 may be insufficient to inhibit tumor growth and may require high concentrations to elicit therapeutic effects. These issues can be overcome by a novel pan-GLUT inhibitor, Gluor, which targets the glucose transporters GLUT-1, GLUT-2, and GLUT-3, with antitumor effects at nanomolar concentrations. However, detailed investigations of the effect of Gluor *in vivo* are limited (230, 231).

### 4.3 Targeting lactate transporters

As the predominant lactate transporters in tumor cells, MCT1 and MCT4 prevent intracellular acidification and maintain normal tumor cell growth. Consequently, MCT inhibitors have great potential for clinical applications in antitumor treatments. α-Cyano-4-hydroxycinnamate (CHC) is a selective and reversible inhibitor of MCT1 that can significantly inhibit tumor growth and reverse hypoxic conditions in the TME (7). Choi et al. effectively inhibits tumor growth by reducing MCT4 expression levels in tumor-bearing nude mice treated with an MCT4-targeting antisense oligonucleotide (MCT4 ASO) (232). Various potent MCT1 inhibitors have been reported, including SR13800, AZD3965, BAY-8002, and 7ACC2 (233). AZD3965 is an orally bioavailable MCT1 inhibitor in phase I clinical trials that effectively increases T cell infiltration, reduces lactate efflux from tumor cells, and reverses the immunosuppressive microenvironment of solid tumors (234). Moreover, syrosingopine has dual inhibitory effects on MCT1 and MCT4. In combination with metformin, which inhibits oxidative phosphorylation, sylrosiglitazone induces significant cytotoxic reactions and reduces the effective concentration of metformin (235)

### 4.4 Targeting amino acid transporters

Overexpression of CD98, an integral subunit of system x_c_^−^, is often closely associated with poor prognosis in many cancers. The humanized monoclonal antibody IGN523, which targets CD98, has been reported to have significant antitumor activity, and a phase I clinical trial of this antibody in patients with acute myeloid leukemia is currently underway (236). xCT, another functional subunit of the x_c_^−^ transport system, affects intracellular redox homeostasis by regulating GSH metabolism. The transcription factor Nrf2 controls the antioxidant pathway in eukaryotic cells by regulating the expression of SLC7A11 (237). Therefore, the Nrf2 inhibitor triptolide disrupts GSH synthesis in tumor cells and is synthetically lethal in malignant tumors with IDH1 mutations (237). Many studies of inhibitors targeting system x_c_^−^ combined with other therapeutic approaches are also currently underway. Clinical trials of an FDA-approved xCT inhibitor, sulfasalazine (SAS), have been conducted in patients with recurrent glioblastoma. However, a recent study on melanoma indicated that xCT inhibition by SAS allows tumor cells to secrete PD-L1 via exosomes, inducing the M2 polarization of macrophages and leading to the development of resistance to anti-PD-1/PD-L1 therapy (238). Therefore, more detailed studies on the effects of SLC7A11 inhibitors as combination drugs in antitumor immunotherapy are required.

ASCT2-mediated glutamine uptake is inseparable from tumor cell growth and metabolism. GPNA was initially recognized as a commercially available competitive inhibitor of ASCT2, and has since been used in many cancer model studies. However, GPNA is not highly selective for ASCT2 and previous studies have shown that LAT1 and LAT2 are also inhibited by GPNA (239). V-9302 is considered an efficient ASCT2 competitive antagonist with significant inhibitory effects on tumor cell growth and proliferation, both *in vivo* and *in vitro*, while also inducing tumor cell death and oxidative stress (240). Nonetheless, follow-up studies have found that V-9302 has the same inhibitory function on SNAT2 and LAT1 (241). Therefore, specific ASCT2 inhibitors need to be further developed. Recently, monoclonal antibody (mAb) therapies targeting ASCT2 have been preliminarily studied. Suzuki et al. isolated three specific anti-ASCT2 mAbs, KM4008, KM4012, and KM4018, from CHO cells expressing ASCT2, and these effectively inhibited colorectal tumor growth *in vitro* (242). Another humanized anti-ASCT2 mAb, KM8094, exerts antitumor effects in gastric cancer patient-derived xenograft models, indicating its potential application in gastric cancer treatment (243).

L-type amino acid transporters are responsible for the uptake of the large neutral amino acids required for cell growth. By evaluating the antitumor effects of multiple anti-LAT1 mAbs, one study demonstrated that a novel anti-LAT1 mAb effectively inhibited tumor development and thus, it holds promise for cancer therapy (244). The small-molecule LAT1 inhibitor, JPH203, inhibits the growth of multiple solid tumors. Current phase I clinical trials of JPH203 suggest promising applications for the treatment of advanced biliary tract cancer (245).

Immune checkpoint blockade therapy can also be an effective cancer treatment by affecting transporter functions. For instance, immune checkpoint factors PD-1 and CTLA4 trigger immunosuppressive signaling pathways to inhibit the expression of the glucose transporter, GLUT1, and the glutamine transporters, SNAT1 and SNAT2, in T cells, thus limiting nutrient uptake and T cell activation, indicating that mAb drugs targeting immune checkpoints can restore T cell function (246).

### 4.5 Targeting nucleoside transporters

Currently, there is limited published information regarding nucleoside transporter inhibitors. As a selective ENT inhibitor, NBMPR has been widely used in physiological and pharmacological studies of ENT function (247). The FDA-approved antiplatelet drug, ticagrelor, can inhibit ENT1 activity in human erythrocytes and platelets (248, 249). Rapamycin, another potent ENT1 inhibitor, interacts with FKBP to exert its biological function. The efficacy of rapamycin therapy at affecting cellular adenosine uptake and subsequent adenosine signaling suggests a potential new therapeutic strategy for regulating cellular adenosine signaling (250). The fused-pyrimidine nucleoside analog, thienopyrimidine 2′-deoxynucleoside (dMeThPmR) and its ribonucleoside analog (MeThPmR) are inhibitors with high affinity for hCNT1 (251). In colorectal cancer, the histone deacetylase inhibitor trichostatin A (TSA) effectively reverses the downregulation of CNT2 expression mediated by histone deacetylation and enhances the sensitivity of colorectal cancer cells to the chemotherapeutic drug cladribine (196).

## 5 Summary and prospects

Taken together, changes in the expression levels of transporters in the TME are important in regulating tumor progression and immune responses. In the TME, peculiar metabolic pathways and metabolites are major barriers to antitumor immunotherapy, as they affect cellular metabolic activity by regulating the expression of transporters. Tumor proliferation and invasion, as well as immune cell functions, are inseparable from transporter-mediated substrate transport. Current studies on transporter-targeted antitumor drugs have made some progress; however, the evaluation of drug efficacy has mainly focused on tumor cells rather than on the regulation of transporter expression in immune cells. Therefore, more detailed investigations of targeted drugs for selective cytotoxicity, specific inhibitory activity, and sensitivity to metabolites in the TME are required.

Furthermore, changes in metabolite abundance and immune cell activity in the complex and dynamic TME should be considered when describing the effects of changes in transporter expression levels on tumor progression. Metabolism-related genes are currently used to assess immunotherapy efficacy and patient prognosis (252–254). Whether the expression levels of transporters on immune cells can also be used as prognostic indicators for tumor immunotherapy requires further study.

Transporter-targeted drugs combined with chemoradiotherapy and immunotherapy have been shown to have good clinical application potential. Future research should be directed toward the discovery of additional transporters as biomarkers or therapeutic targets, and existing clinical cancer treatments should be optimized by exploring different combination regimens. Rational strategies for tumor therapy may also focus on cellular metabolic features mediated by changes in transporter expression levels in immune cells, to reverse the immunosuppressive TME, thereby enabling more effective antitumor immune responses.

## Author contributions

LY and LC conceived, designed, and supervised the project. LC and YW wrote the manuscript. LC, YW, QH, YL, XQ, ZT, HH, NL, SZ and LY provided critical discussion and revised the manuscript. All authors contributed to the article and approved the submitted version.
